# Cumulative Effects of Prior Concussion and Primary Sport Participation on Brain Morphometry in Collegiate Athletes: A Study From the NCAA–DoD CARE Consortium

**DOI:** 10.3389/fneur.2020.00673

**Published:** 2020-07-28

**Authors:** Benjamin L. Brett, Samuel A. Bobholz, Lezlie Y. España, Daniel L. Huber, Andrew R. Mayer, Jaroslaw Harezlak, Steven P. Broglio, Thomas W. McAllister, Michael A. McCrea, Timothy B. Meier, CARE Consortium Investigators

**Affiliations:** ^1^Department of Neurosurgery, Medical College of Wisconsin, Milwaukee, WI, United States; ^2^Department of Neurology, Medical College of Wisconsin, Milwaukee, WI, United States; ^3^The Mind Research Network/Lovelace Biomedical and Environmental Research Institute, Albuquerque, NM, United States; ^4^Neurology and Psychiatry Departments, University of New Mexico School of Medicine, Albuquerque, NM, United States; ^5^Department of Psychology, University of New Mexico, Albuquerque, NM, United States; ^6^Department of Epidemiology and Biostatistics, Indiana University, Bloomington, IN, United States; ^7^School of Kinesiology and Michigan Concussion Center, University of Michigan, Ann Arbor, MI, United States; ^8^Department of Psychiatry, Indiana University School of Medicine, Bloomington, IN, United States; ^9^Department of Biomedical Engineering, Medical College of Wisconsin, Milwaukee, WI, United States; ^10^Department of Cell Biology, Neurobiology and Anatomy, Medical College of Wisconsin, Milwaukee, WI, United States

**Keywords:** concussion and sports, traumatic brain injury, CARE consortium, brain morphometry, contact sport exposure, grey matter (GM)

## Abstract

Prior studies have reported long-term differences in brain structure (brain morphometry) as being associated with cumulative concussion and contact sport participation. There is emerging evidence to suggest that similar effects of prior concussion and contact sport participation on brain morphometry may be present in younger cohorts of active athletes. We investigated the relationship between prior concussion and primary sport participation with subcortical and cortical structures in active collegiate contact sport and non-contact sport athletes. Contact sport athletes (CS; *N* = 190) and matched non-contact sport athletes (NCS; *N* = 95) completed baseline clinical testing and participated in up to four serial neuroimaging sessions across a 6-months period. Subcortical and cortical structural metrics were derived using FreeSurfer. Linear mixed-effects (LME) models examined the effects of years of primary sport participation and prior concussion (0, 1+) on brain structure and baseline clinical variables. Athletes with prior concussion across both groups reported significantly more baseline concussion and psychological symptoms (all *p*s < 0.05). The relationship between years of primary sport participation and thalamic volume differed between CS and NCS (*p* = 0.015), driven by a significant inverse association between primary years of participation and thalamic volume in CS (*p* = 0.007). Additional analyses limited to CS alone showed that the relationship between years of primary sport participation and dorsal striatal volume was moderated by concussion history (*p* = 0.042). Finally, CS with prior concussion had larger hippocampal volumes than CS without prior concussion (*p* = 0.015). Years of contact sport exposure and prior concussion(s) are associated with differences in subcortical volumes in young-adult, active collegiate athletes, consistent with prior literature in retired, primarily symptomatic contact sport athletes. Longitudinal follow-up studies in these athletes are needed to determine clinical significance of current findings.

## Introduction

Recent reports have generated mixed results on whether prior sport-related concussion (SRC) or head impact exposure without SRC may negatively affect brain structure and function and result in adverse long-term neurobehavioral outcomes (1–6). Previous studies have reported differences in brain structure in retired athletes associated with prior concussion or indirect measures of repetitive head impacts (RHIs), such as multiple years of contact sport participation and/or the age of first participation of contact sport (i.e., age at first exposure). RHI differs from SRC in that they involve head impacts below the relative magnitude of concussive injury (i.e., absence of acute symptoms) and some have suggested that these different mechanisms of insult may have differing effects on long-term outcomes (2, 7–10). Prior reports of smaller hippocampal and amygdala volumes as well as thinner frontal and temporal cortices have been recorded in retired athletes in relation to exposure factors or prior concussion relative to either former non-contact athletes or non-athlete controls (11–16). Similarly, thalamic volumes have been inversely associated with level of exposure to contact sport in retired professional football players (17). Conversely, other studies have reported no differences in brain morphometry among former contact sport athletes with different competition level participation histories (18–20). Prior work, however, did not aim to separate the effects of exposure to contact sport from the long-term effects of concussion, did not account for other confounding factors, or focused on older adults who were typically many years removed from their most recent concussion or contact sport exposure (21).

There is emerging evidence to suggest that similar effects of prior concussion and contact sport participation on brain morphometry may be present in younger cohorts of active athletes, with evidence of smaller hippocampal volume and thinner cortex in contact sport athletes and mixed martial artists in relation to exposure variables, prior concussion, or baseline post-concussion symptoms (22–26). Nevertheless, the extent to which prior concussion and exposure to contact sport participation independently affect cortical and subcortical structure in young-adult, active athletes has not been definitively determined.

The goal of this project from the National Collegiate Athletic Association (NCAA)–Department of Defense (DoD) Grand Alliance: Concussion Assessment, Research, and Education (CARE) Consortium was to determine the effects of prior concussion and exposure history on subcortical and cortical structures in active collegiate contact and non-contact sport athletes. We tested the hypothesis that prior concussion and years of primary contact sport participation would be inversely associated with subcortical volume (i.e., thalamus, amygdala, dorsal striatum, and hippocampus) as well as cortical thickness and volume (e.g., frontal and temporal cortices).

## Methods

### Participants

This study was approved by the Medical College of Wisconsin Institutional Review Board and the Human Research Protection Office (HRPO). The study design and methods of NCAA–DoD CARE Consortium have been described elsewhere (27). Data for this study from the CARE Consortium are publicly available from the National Institute of Health (NIH) Federal Interagency Traumatic Brain Injury (TBI) Research (FITBIR) Informatics System (https://fitbir.nih.gov). The current study includes athletes enrolled at baseline at four sites where the advanced neuroimaging protocol is deployed as part of the CARE Advanced Research Core (ARC): University of North Carolina at Chapel Hill (UNC), University of Wisconsin-Madison (UW), University of California Los Angeles (UCLA), and Virginia Tech (VT). All participants provided written informed consent. Concussed contact sport athletes (i.e., football, hockey, lacrosse, and soccer) participated in up to four neuroimaging sessions including at 24–48 h post-injury (i.e., 24–48 h), following clearance to begin the return-to-play (RTP) progression (i.e., asymptomatic), 7 days following unrestricted RTP (i.e., post-RTP), and 6 months post-injury (i.e., 6 months). Staff at each institution diagnosed concussions using a standard definition as previously described (27, 28). Non-injured contact sport athletes participated in similar follow-up visits and were matched to injured athletes based on institution, sport, gender, race/ethnicity, estimate of premorbid verbal intellectual functioning (i.e., Wechsler Test of Adult Reading; WTAR), concussion history, years of primary sport participation, status as a starter, and head impact exposure estimate (or data when available). For the current study focused on the cumulative effects of exposure and prior concussion, injured and non-injured contact sport athletes were collapsed into a single group of contact sport athletes (CS; *N* = 211). Non-contact sports athletes (i.e., baseball, basketball, cross-country/track, field event, and softball) matched on institution, gender, race/ethnicity, and estimate of premorbid verbal intellectual functioning completed similar neuroimaging visits and served as controls for the current study (NCS; *N* = 101). Non-contact control athletes were not excluded if they had prior contact sport exposure. Of the 312 athletes with imaging and clinical data available, 27 were excluded due to additional exclusion criteria for the current study that included history of moderate/severe traumatic brain injury or seizure disorder, acute trauma-related findings on MRI identified during a clinical overread by radiologist, poor scan quality or processing errors, or missing data for primary variables of interest. A final total of 190 CS and 95 NCS athletes were included in analyses.

### Baseline Clinical Battery

Demographic and health history information were collected at baseline, prior to the competitive season, and at follow-up visits. Athletes self-reported the number of prior concussions (diagnosed and undiagnosed) and years of exposure in their primary sport. The current study employed a unitary definition criteria for the diagnosis of concussion across all sites. Specifically, as a standard clinical definition of concussion based on the US Department of Defense (27, 28), the CARE consortium operationally defined a concussion as “a change in brain function following a force to the head, which may be accompanied by a temporary loss of consciousness, but is identified in awake individuals with measures of neurologic and cognitive dysfunction.” The self-reported number of years of primary sport participation served as a proxy to sport exposure for both groups. The WTAR was collected at baseline and used to estimate pre-morbid intelligence. Additional measures collected at baseline included severity of symptoms commonly associated with concussion [Sport Concussion Assessment Tool−3rd Edition symptom checklist (SCAT)], psychological symptomology [Brief Symptom Inventory-18 Global Severity Index (BSI-GSI)], postural stability [Balance Error Scoring System (BESS)], and cognition [Standardized Assessment of Concussion (SAC)].

### Imaging Protocol and Processing

High-resolution T1-weighted images (1 × 1 × 1 mm) were acquired on 3T MRI scanners at each site. Specifically, a 3D magnetization-prepared rapid gradient-echo sequence was collected on Siemens MAGNETOM Prisma (32-channel head coil) and MAGNETOM Trio (32- or 12-channel head coils) scanners at UNC and UCLA with the following parameters: TR/TE/TI = 2,300/2.98/900 ms, flip angle = 9°, FOV = 256 mm, matrix = 256 × 256, 176 slices. A 3D magnetization-prepared rapid gradient-echo sequence was collected on the Siemens MAGNETOM Trio (8-channel head coil) scanner at VT with the following parameters: TR/TE/TI = 2300/2.89/900 ms, flip angle = 9°, FOV = 256 mm, matrix = 256 × 256, 176 slices. A 3D Brain Volume (BRAVO) sequence was collected on a General Electric Discovery MR750 scanner with the following parameters: TR = 6.62 to 6.652, TE = 2.91 to 2.928, TE = 450 ms, flip angle = 12°, FOV = 256 mm, matrix = 256 × 256, 164 slices.

Cortical surfaces were reconstructed and tissue segmentation was performed on the T_1_-weighted images using the following steps implemented in FreeSurfer v5.3: removal of non-brain tissue, automated Talairach transformation, segmentation of the subcortical white matter and deep gray matter structures, intensity normalization, tessellation of the gray/white matter boundary, automated topology correction, and surface deformation following the intensity gradients of the image in order to accurately place the boundaries between different tissue types. The longitudinal stream was used to extract reliable volume and thickness estimates (29). Visual inspection of each step of the processing stream was conducted and scans with poor quality (motion artifacts, poor tissue contrast, segmentation issues, etc.) were excluded, as described above. Cortical surface metrics, including cortical thickness and cortical volume, were calculated and smoothed with a full-width half-maximum of 10 mm.

### Statistical Analysis

Statistical analyses were conducted using SPSS version 24 unless otherwise indicated. Independent samples *t*-tests, chi-square tests, and Fisher Exact tests were used to compare demographic variables between groups (NCS vs. CS). Generalized linear models were used to determine the effects of contact sport status (i.e., CS vs. NCS), sport exposure (i.e., years of primary sport participation; mean centered), prior concussion, and the interaction of contact sport status and years participation on baseline clinical measures. Only 5.96% had a history of 2 prior concussions and 1.75% of included participants had 3 or more prior concussions; thus, prior concussion was treated as a binary factor (coded as 0, 1+). Sensitivity analyses showed that modeling concussion history as categorical (0, 1, and 2+) did not meaningfully alter results of our executed analyses. Age, sex, race, ADHD diagnosis, and body mass index (BMI) were included as *a priori* covariates. BSI-GSI, BESS, and SCAT scores were modeled using negative binomial distributions. For subcortical volumes, *a priori* analyses focused on bilateral thalamus, hippocampus, amygdala, and dorsal striatum (i.e., combined caudate and putamen) due to their vulnerability to brain injury (16, 17, 22, 23, 30). Linear mixed-effects (LME) models were fit to assess the effects of prior concussion, sport exposure, contact sport status, and the interaction between contact sport (yes or no) and exposure on structural measures, with visit modeled as a categorical variable to account for repeating scanning over time and participant modeled as a random effect. Estimated intracranial volume (or mean thickness for cortical thickness analyses), age, sex, race, ADHD diagnosis, and BMI were included as covariates. A combined covariate accounting for site, scanner, and head coil was also included.

Previously published studies and ongoing work from the CARE Consortium demonstrate minimal to no acute effects of a recent single concussion on gray matter structural metrics (31, 32). Therefore, concussed athletes with and without acute injury were collapsed into a single group. Sensitivity analyses showed that treating contact sport groups separately had no effect on results. Significant interactions of primary sport participation and group (CS vs. NCS) were followed-up with similar LME models fit within each group separately. Additional LME models were fit with each contact sport separately to determine the association between years of primary sport participation and structural metrics, as indicated (see Results). Finally, LME models were fit in CS athletes to identify potential interactions between years of primary contact sport participation and prior concussion on structural measures using the same covariates identified above in addition to prior concussion (coded as 0, 1+), years of contact sport exposure, and their interaction.

Similar LME models as described above were fit for vertex-wise analyses of cortical surface metrics (i.e., cortical thickness and volume) using FreeSurfer's spatiotemporal mass-univariate model (33, 34). This involved segmenting regions with homogenous covariance parameters from initial estimates and fitting the model based on the generated regions, which allows for increased power over a standard mass-univariate approach while controlling for the false discovery rate (FDR). A two-stage linear step-up FDR correction was used for multiple comparisons correction for surface analyses. Independent variables of interest included sport group (CS vs. NCS), number of prior concussions, the group by years of primary sport participation interaction, and the interaction of prior concussion by years of primary sport participation. A nominal significance alpha of 0.05 was used to determine significant effects.

## Results

### Demographics and Baseline Clinical Measures

**Figure 1:**
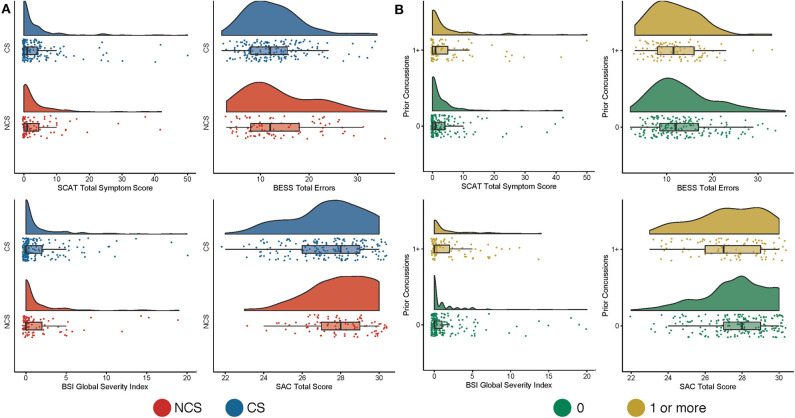
Baseline clinical data based on sport and prior concussion. Shown are raincloud plots of clinical data at baseline contact sport (CS) and non-contact sport (NCS) athletes **(A)** and in participants without (0) and with at least one prior concussion (1+; **B**).

**Figure 2:**
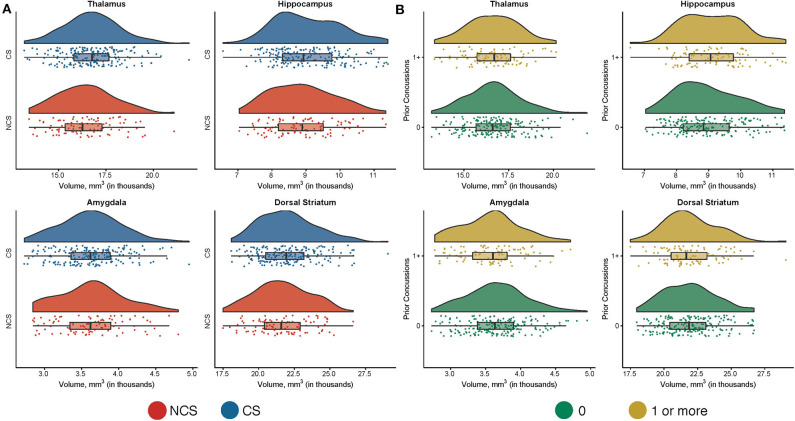
Subcortical volumes based on sport and prior concussion in whole sample. Shown are raincloud plots of subcortical volumes in contact sport (CS) and non-contact sport (NCS) athletes **(A)** and in participations without (0) and with at least one prior concussion (1+; **B**). The mean subcortical volumes across all visits are displayed for individual participants.

Demographic information and medical history of CS and NCS are provided in Table 1. CS and NCS athletes had significant group differences in BMI, race, ethnicity, prior concussion, and presence of ADHD (*p*s < 0.05). Statistics for the independent variables of interest across the entire sample are presented in Table 2. GLM showed that athletes with prior concussion reported significantly higher SCAT (*p* < 0.01) and psychological (i.e., BSI-GSI) symptom scores at baseline (Figures 1, 2; *p* < 0.05). There were no effects of year of primary sport participation or interactions of sport by years of participation on baseline clinical measures (*p*s > 0.05).

**Table 1:** Sample characteristics.

**Demographics**	**Non-contact athletes**	**Contact athletes**	**Statistic**
Total No.	95	190	
Sex (No. Male)	77	153	χ^2^(1), *p* = 0.92
Race			Fisher's Exact, *p* = 0.02
Black or African American (No.)	12	54	
White (No.)	72	121	
Other (No.)	9	12	
Unknown/NR (No.)	2	3	
Ethnicity			χ^2^(2) = 10.25, *p* = 0.006
Hispanic (No.)	7	10	
Non-hispanic (No.)	87	157	
Unknown/NR (No.)	1	23	
Age at first scan, M (SD)	20.52 (1.23)	20.27 (1.28)	*t*(283) = 1.62, *p* = 0.10
Body mass index, M (SD)	23.48 (3.17)	26.8 (5.03)	*t*(283) = −5.88, *p* < 0.001
WTAR standard score, M (SD)	109.16 (12.59)	108.31 (13.32)	*t*(234) = 0.40, *p* = 0.69
Years participation, M (SD)	11.39 (3.62)	11.63 (3.63)	*t*(283) = −0.53, *p* = 0.60
Sport			
Football (No.)	NA	109	
Ice hockey (No.)	NA	17	
Lacrosse (No.)	NA	13	
Soccer (No.)	NA	51	
Baseball (No.)	34	NA	
Basketball (No.)	11	NA	
Cross-country/track (No.)	36	NA	
Field event (No.)	8	NA	
Softball (No.)	6	NA	
Previous concussions			χ^2^(1) = 15.90, *p* < 0.001
0 (No.)	78	111	
1 or more (No.)	17	79	
Scanner/Coil/Site			Fisher's Exact, *p* = 0.50
UW GE MR750 (No.)	28	59	
VT siemens Trio 8ch (No.)	7	15	
UNC siemens Prisma 32ch (No.)	25	39	
UNC siemens Trio 12ch (No.)	0	1	
UNC siemens Trio 32ch (No.)	8	25	
UCLA siemens Prisma 32ch (No.)	24	35	
UCLA siemens Trio 12ch (No.)	2	10	
UCLA siemens Trio 32ch (No.)	1	6	
Clinical history			
ADHD (No.)	2	20	χ^2^(1) = 6.30, *p* = 0.012
Migraines (No.)	4	15	χ^2^(1) = 1.38, *p* = 0.24
Psychiatric disorder (No.)	6	6	Fisher's exact, *p* = 0.22
Learning disorder (No.)	2	4	Fisher's exact, *p* = 1
Memory disorder (No.)	0	5	Fisher's exact, *p* = 0.17
Balance disorder (No.)	0	1	Fisher's exact, *p* = 1
Sleep disorder (No.)	1	2	Fisher's exact, *p* = 1
Meningitis (No.)	0	2	Fisher's exact, *p* = 0.55
Diabetes (No.)	0	2	Fisher's exact, *p* = 0.55
Hearing problems (No.)	0	2	Fisher's exact, *p* = 0.55
Vision problems (No.)	0	1	Fisher's exact, *p* = 1

*No., number; NR, not reported; WTAR, Wechsler Test of Adult Reading; UW, University of Wisconsin-Madison; VT, Virginia Tech; UNC, University of North Carolina at Chapel Hill; UCLA, University of California Los Angeles; GE, General Electric; ch, channel; ADHD, attention deficit hyperactivity disorder; NA, not applicable*.

**Table 2:** Statistics for generalized linear models and linear mixed effects models in whole sample.

	**Statistic**	**Est**.	**Std. Err**.	***p*-value**	**95% CI [lower, upper]**
**SCAT SYM. SEV**.					
Sport (NCS vs. CS^a^)	Wald χ^2^(1) = 0.60	−0.13	0.17	0.44	[−0.47, 0.20]
Prior Con. (0 vs. 1+^b^)	Wald χ^2^(1) = 9.05	0.45	0.15	*0.003*	[0.16, 0.73]
Sport by Yrs. Part.	Wald χ^2^(1) = 0.001	0.001	0.05	0.99	[−0.10, 0.10]
**BESS**					
Sport (NCS vs. CS^a^)	Wald χ^2^(1) = 0.26	0.08	015	0.61	[−0.22, 0.37]
Prior Con. (0 vs. 1+^b^)	Wald χ^2^(1) = 0.36	−0.08	0.14	0.55	[−0.36, 0.19]
Sport by Yrs. Part.	Wald χ^2^(1) = 0.12	0.02	0.04	0.73	[−0.07, 0.10]
**SAC**					
Sport (NCS vs. CS^a^)	Wald χ^2^(1) = 3.69	0.49	0.26	0.06	[−0.10, 0.98]
Prior Con. (0 vs. 1+^b^)	Wald χ^2^(1) = 0.63	−0.18	0.23	0.43	[−0.63, 0.27]
Sport by Yrs. Part.	Wald χ^2^(1) = 2.67	0.11	0.07	0.10	[−0.02, 0.24]
**BSI-GSI**					
Sport (NCS vs. CS^a^)	Wald χ^2^(1) = 0.44	0.13	0.19	0.51	[−0.25, 0.50]
Prior Con. (0 vs, 1+^b^)	Wald χ^2^(1) = 5.92	0.43	0.18	*0.02*	[0.08, 0.78]
Sport by Yrs. Part.	Wald χ^2^(1) = 0.004	0.003	0.06	0.95	[−0.11, 0.12]
**THALAMUS**					
Sport (NCS vs. CS^a^)	*F*_(1, 273.24)_ = 0.48	−97.63	141.34	0.49	[−375.89, 180.62]
Prior Con. (0 vs. 1+^b^)	*F*_(1, 272.48)_ = 0.10	39.74	128.01	0.76	[−291.75, 212.27]
Sport by Yrs. Part.	*F*_(1, 504.14)_ = 6.74	82.31	31.70	*0.010*	[20.03, 144.59]
**HIPPOCAMPUS**					
Sport (NCS vs. CS^a^)	*F*_(1, 274.90)_ = 0.03	17.90	96.61	0.85	[−172.29, 208.10]
Prior Con. (0 vs. 1+^b^)	*F*_(1, 272.31)_ = 1.99	123.74	87.63	0.16	[−48.77, 296.25]
Sport by Yrs. Part.	*F*_(1, 755.46)_ = 3.38	33.55	18.24	0.07	[−2.26, 69.35]
**AMYGDALA**					
Sport (NCS vs. CS^a^)	*F*_(1, 268.83)_ = 1.57	63.02	50.24	0.21	[−35.90, 161.94]
Prior Con. (0 vs. 1+^b^)	*F*_(1, 268.98)_ = 0.58	−34.56	45.50	0.45	[−124.14, 55.02]
Sport by Yrs. Part.	*F*_(1, 435.23)_ = 0.13	−4.24	11.75	0.72	[−27.32, 18.85]
**DORSAL STRIATUM**					
Sport (NCS vs. CS^a^)	*F*_(1, 275.51)_ = 0.04	−43.20	214.02	0.84	[−464.51, 378.12]
Prior Con. (0 vs. 1+^b^)	*F*_(1, 272.79)_ = 1.98	272.95	194.16	0.16	[−109.29, 655.19]
Sport by Yrs. Part.	*F*_(1, 785.01)_ = 0.23	19.04	39.33	0.63	[−58.17, 96.25]

a *Reference group is CS*.

b *Reference group is 0, Est., estimate; Std. Err., standard error; CI, confidence interval; SCAT, Sport Concussion Assessment Tool; Sym., symptom; Sev., severity; BESS, Balance Error Scoring System; SAC, Standardized Assessment of Concussion; BSI-GSI, Brief Symptom Inventory-18 Global Severity Index; Yrs. Part., Years of primary sport participation; Con., concussion. Significant p values are italicized and underlined and represent results from the omnibus F tests*.

### Structural Measures—Whole Sample

**Figure 3:**
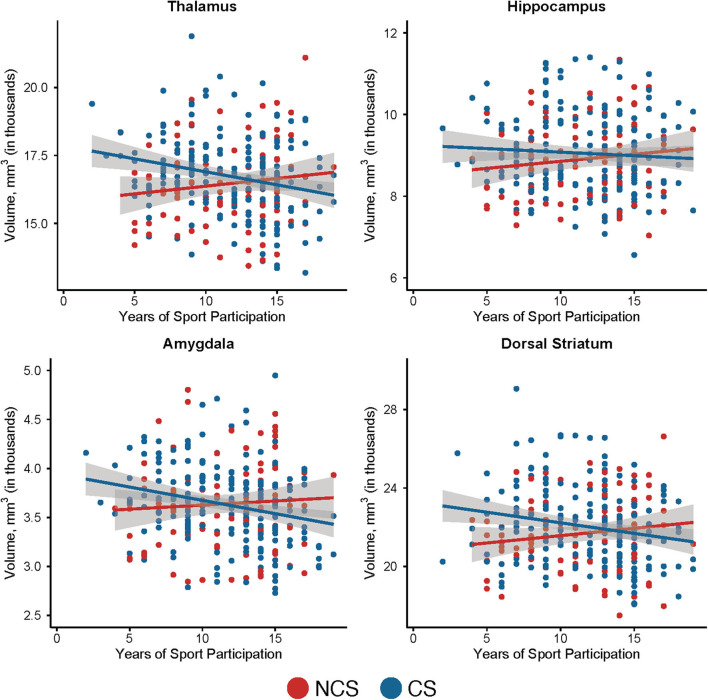
Relationship between subcortical volumes and years of primary sport participation in contact and non-contact sport athletes. Shown are scatter plots of years of primary sport participation vs. subcortical volume in non-contact sport (NCS) and contact sport (CS) athletes with 95% confidence intervals. The mean subcortical volumes across all visits are displayed for individual participants.

There were no significant differences in subcortical volume, cortical volume, or cortical thickness between CS and NCS athletes (Figure 1; *p*s > 0.05) or between athletes with and without prior concussion (Figure 2; *p*s > 0.05). Type of primary sport participation (i.e., CS vs. NCS), however, moderated the relationship between years of primary sport participation and thalamic volume (*p* = 0.01; Table 2). Follow-up tests showed a significant inverse association between thalamic volume and primary sport participation in CS athletes, *F*_(1, 227.62)_ = 7.46, *p* = 0.007, *B* = −60.09, SE = 21.99 (95% CI: −103.43, −16.75), while the relationship between volume and sport participation was not significant in NCS athletes, *F*_(1, 129.73)_ = 1.70, *p* = 0.20, *B* = 40.11, SE = 30.81 (95% CI: −20.84, 101.05; Figure 3). The group by years of participation interaction was not significant for amygdala, dorsal striatal, or hippocampal volume, or cortical thickness or volume (*p*s > 0.05).

Follow-up analyses were conducted to determine if the inverse association between contact sport participation and thalamic volumes was driven by a particular contact sport. Due to small numbers, ice hockey and lacrosse athletes were grouped together. There was a non-significant inverse association between thalamic volume and years of primary sport participation in football players, *F*_(1, 118.93)_ = 3.09, *p* = 0.08, *B* = −56.64, SE = 32.22 (95% CI: −120.4, 7.16) and a non-significant inverse association between volume and years of primary sport participation in soccer players, *F*_(1, 71.04)_ = 3.13, *p* = 0.08, *B* = −89.96, SE = 50.82 (95% CI: −191.30, 11.37). There was also a non-significant positive association between primary sport participation and volume in the combined ice hockey and lacrosse group, *F*_(1, 33.75)_ = 2.14, *p* = 0.15, *B* = 91.58, SE = 62.61 (95% CI: –.35.72, 218.88).

### Interaction of Primary Contact Sport Participation and Prior Concussion

**Figure 4:**
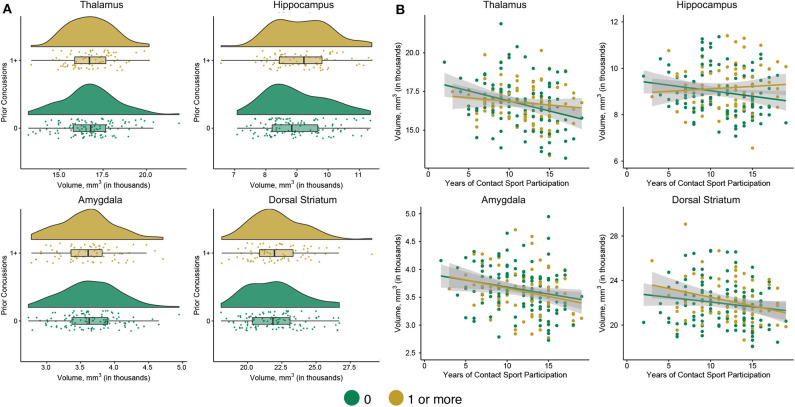
Subcortical volumes based on prior concussion and association with years of primary sport participation in contact sport athletes only. **(A)** Shown are raincloud plots of subcortical volumes in participants without (0) and with at least one prior concussion (1+). **(B)** Shown are scatter plots of years of primary sport participation vs. subcortical volume in contact sport athletes without (0) and with at least one prior concussion (1+) with 95% confidence intervals. The mean subcortical volumes across all visits are displayed for individual participants.

Statistics for the independent variables of interest for analyses in CS athletes only are presented in Table 3. CS athletes with prior concussion had significantly larger hippocampal (Figure 4; *p* = 0.016) and dorsal striatal (*p* = 0.041) volumes than those without prior concussion. Amygdala volume, thalamic volume, cortical volume, and cortical thickness did not differ in CS with and without prior concussion (*p*s > 0.05). The interaction of prior concussion and years of primary contact sport participation was significant for dorsal striatal volume (*p* = 0.041). Follow-up analyses showed that contact sport exposure was non-significantly associated with dorsal striatal volume in CS athletes with prior concussion, *F*_(1, 145.48)_ = 2.2394, *p* = 0.105, *B* = −83.07, SE = 50.91 (95% CI: −183.69, 17.54.49), while years of exposure was not associated with volume in CS athletes without prior concussion, *F*_(1, 188.44)_ = 0.00, *p* = 0.99, *B* = −0.06, SE = 39.67 (95% CI: −78.31, 78.19). The interaction of prior concussion and years of primary contact sport participation was not significant for any other structural measure (*p*s > 0.05).

**Table 3:** Statistics for linear mixed effects models for subcortical structures in contact sports athletes only.

	**Statistic**	**Est**.	**Std. Err**.	***p***	**95% CI [lower, upper]**
**THALAMUS**					
Yrs. Part.	*F*_(1, 226.80)_ = 7.05	−53.38	29.81	*0.008*	[−112.10, 5.34]
Prior Con. (0 vs 1+^a^)	*F*_(1, 178.08)_ = 0.52	103.48	142.89	0.47	[−178.49, 385.45]
Prior Con. by Yrs. Part.	*F*_(1, 288.60)_ = 0.11	11.33	34.10	0.74	[−55.78, 78.46]
**HIPPOCAMPUS**					
Yrs. Part.	*F*_(1, 297.65)_ = 0.01	6.22	18.03	0.94	[−29.24, 41.69]
Prior Con. (0 vs. 1+^a^)	*F*_(1, 177.88)_ = 5.91	231.20	95.13	*0.016*	[43.48, 418.93]
Prior Con. by Yrs. Part.	*F*_(1, 422.90)_ = 0.28	−10.48	19.85	0.60	[−28.53, 49.50]
**AMYGDALA**					
Yrs. Part.	*F*_(1, 211.24)_ = 0.03	−11.91	11.02	0.87	[9.79, 33.62]
Prior Con. (0 vs 1+^a^)	*F*_(1, 173.15)_ = 0.00	0.66	51.99	0.99	[−103.28, 101.96]
Prior Con by Yrs. Part.	*F*_(1, 260.97)_ = 2.77	−21.10	12.69	0.10	[−46.08, 3.89]
**DORSAL STRIATUM**					
Yrs. Part.	*F*_(1, 349.06)_ = 2.69	−95.73	40.41	0.10	[−175.17, −16.28]
Prior Con. (0 vs 1+^a^)	*F*_(1, 179.05)_ = 4.24	466.42	226.47	*0.041*	[−19.53, 913.32]
Prior Con. by Yrs. Part.	*F*_(1, 491.30)_ = 4.20	−89.31	43.59	*0.041*	[−174.95, −3.67]

a *Reference group is 0. Est, estimate; Std. Err., standard error; CI, confidence interval; Yrs. Part., Years of primary sport participation; Con., concussion. Significant p values are italicized and underlined and represent results from the omnibus F-tests*.

## Discussion

The current study investigated the effects of contact sport exposure and concussion history on cortical and subcortical structural measures in a large sample (*N* = 285) of active collegiate athletes. At baseline, athletes with prior concussion reported significantly more SCAT and psychological symptoms. Although there were no differences in brain structure between contact and non-contact athletes, the relationship between the number of years of primary sport participation and volume in specific subcortical structures differed in the two groups. Specifically, there was a significant inverse relationship between thalamic volume and years of primary sport participation in contact but not non-contact athletes, an effect largely driven by soccer and football athletes. Contact sport athletes with prior concussion had larger hippocampal and dorsal striatal volumes than those without prior concussion. No effects of primary sport participation or prior concussion were observed in the cortex. Although causal relationships cannot be determined, current results suggest that a combination of primary contact sport exposure and concussion history may be associated with differences in subcortical structure in young, active collegiate athletes.

Studies investigating the effects of prior concussion on baseline symptom reporting have been mixed (35, 36), with some studies reporting similar elevations in baseline symptom endorsement (37–40). The lack of an observed relationship between years of primary sport participation and baseline clinical variables is consistent with large-scaled studies (*N*s > 1,000) that also failed to observe a relationship between age at first exposure to football and similar clinical outcomes in active athletes (40, 41). It is important to note that the differences in SCAT and BSI-GSI symptoms (marginal mean differences = 2.0 and 0.9, respectively) in athletes with and without prior concussion, while statistically significant, are minor and without clinical implications. Nevertheless, the potential relationship between subtle differences in baseline clinical measures in active athletes and the adverse long-term effects documented in retired athletes merits prospective, longitudinal follow-up studies (1, 12, 14–17).

An association between primary contact sport participation (i.e., exposure) and thalamic and/or dorsal striatal volume has been previously reported in other samples of athletes. A negative association between years of sport participation and bilateral thalamic volumes was reported in a sample of 86 symptomatic (cognitive and/or mood) former professional football players (mean age = 54.9) (17). Likewise, fight exposure was inversely associated with thalamus and caudate volumes in a large cohort of professional mixed martial arts fighters and boxers (42). The current results are consistent with this prior work and extend previous findings to active collegiate athletes. Moreover, results suggest a region-specific, additive effect of prior concussion and years of contact sport participation.

In the current study, exposure was quantified by the number of years of primary sport participation with the hypothesis that more years of contact sport is associated with greater exposure to RHIs. It has been hypothesized that RHI rather than frank concussion are associated with negative outcomes in retired athletes (8). Estimated RHI, more so than concussion, significantly predicted impairment on a cognitive screen, as well as self-reported executive dysfunction, depression, apathy, and behavioral dysregulation in 93 former amateur football players (2). Additionally, earlier age of first exposure has also been associated with similar outcomes in former football players with various levels of play experience (mean age = 50.7). Thus, the inverse association between years of primary contact sport participation and volume recorded in the current study is consistent with this hypothesis.

There are, however, several other factors that could explain differential effects of years of primary sport participation in contact vs. non-contact sports, such as temperamental (e.g., risk taking behavior) or lifestyle factors. For example, athletes who elect to play higher risk sports are more likely to engage in risk-taking behaviors (43–45). Thus, the degree to which reductions in thalamic or dorsal striatum volumes are attributable to RHI or some other mechanism is not entirely clear. Additionally, the effect of contact sport exposure on brain morphometry might differ based on the age or developmental period at which the exposure occurs. Future studies are needed to test this hypothesis. Ultimately, future work is needed to compare structural measures with more direct measures of RHI such as head impact sensor telemetry data. Interestingly, the two sports where a trend association between years of primary participation and brain morphometry differences was observed have different head impact profiles within a single season, dependent on age, when measured using impact sensor telemetry systems (46).

In contrast to our hypothesis, there was no significant association between exposure and hippocampal volume. Rather, contact sport athletes with prior concussion had larger hippocampal volumes. Several previous studies in both retired and active athletes have reported smaller hippocampal volumes associated with more exposure or repeated concussion (16, 18, 22, 23). Other studies, however, have reported no associations (13, 19, 20), while a recent study observed larger hippocampal volumes in current athletes with prior concussion relative to matched athletes without a history of prior concussion (47). The current sample's distribution of concussion history (mean of 0.44 concussions) was considerably less than other prior studies recording an association between brain morphometry and concussion history. Prior studies have shown that former athletes with three or more concussions were at the greatest risk of experiencing long-term adverse outcomes (i.e., cognitive impairment and depression), as compared to those with no or one to two prior SRCs (5, 6). Given that only 5 athletes (1.75%) in the current sample reported a history of three or more prior concussions, the prior effects of SRC may have gone undetected.

Similarly, we observed no evidence of differences in cortical structure associated with exposure or prior concussion, despite prior studies reporting differences in cortex in both active and retired athletes. (11–16, 22, 24–26). Others, however, have also failed to record such a relationship or were unable to differentiate the effects of exposure and concussion (18–20). One possible explanation is that subcortical regions experience more mechanical stress from head impacts relative to the cortical mantle (48). The lack of association between years of primary sport participation and cortical structural changes was not entirely consistent with the prevailing theory of regional vulnerability, given that the preferential regions of neuropathology (i.e., CTE) involve the perivascular spaces within the depths of the cortical sulci (7, 49). The relationship between subcortical morphometry changes observed in the current study and regional vulnerability localized to the depths of the cortical sulci is not entirely clear. Further work is needed to understand whether these are two distinct processes potentially contributing to adverse long-term neurobehavioral outcomes, or subcortical morphometry differences contribute to the cortical regional vulnerability through a mechanism such as disruption in the thalamocortical connectivity (50). Ultimately, large-scale, longitudinal studies following athletes across multiple years during and after contact sport exposure are needed to definitively identify the effects or prior concussion on brain structure and behavior.

The clinical significance of the current structural findings is yet to be determined. It is possible that a subset of the athletes with structural differences observed in the current study will experience a slow progression of the structural differences and ultimately experience clinical deficits later in life reported by some studies (1–6). It is also likely that the changes observed in the current study may not have any unique long-term sequelae in isolation; however, coupling with multiple other factors, such as substance (ab)use or chronic stress, may contribute to a cumulative burden that develops into adverse structural and clinical outcomes later in life. Finally, it is also important to consider the observed effects in context of other known factors that impact brain structure, such as obesity (51) or a myriad of other lifestyle and medical factors (52). Given the complexity of this matter due to these and other inherent confounding factors, replication of the current findings is essential in order to be able to draw definitive conclusion.

### Limitations

Years of primary sport participation and the number of prior concussions were based on self-report, which may under- or overestimate true values (53). For this manuscript, years of primary sport participation was selected a priori as the most parsimonious means to estimate RHI or exposure over a career, as this measure aligns with the overall design of the CARE Consortium (i.e., grouping athletes based on primary sport). There are alternative measures that could be used, such as summing the total number of years listed across various sports at different levels of play or indices based on several sport-related factors and published sensor data (2, 54). However, ~70% of collegiate athletes begin to specialize in a single sport during childhood or adolescence (55). Moreover, estimates of cumulative head impact exposure based on sensor data are currently only available in football athletes. Additional research is needed to create a more refined picture of the potential effects of contact sport participation on brain morphometry, such as examination of the effects of exposure at various periods of neurodevelopment. Finally, the CARE protocol was not specifically designed to address the cumulative effects of prior concussion and exposure to contact sport and causal relationships cannot be determined.

### Conclusion

Differences in subcortical volumes (i.e., thalamus and dorsal striatum) associated with years of primary contact sport participation reported elsewhere among older retired athletes were detected in a large cohort of young-adult, currently active collegiate athletes. Prior concussion was also associated with subtle differences in symptom reporting among young-adult, active collegiate athletes (i.e., elevated). Though the long-term implications of these findings remain uncertain, efforts are currently underway to longitudinally follow these athletes to properly understand the underlying mechanism and time frame of these effects and whether they are associated with latent clinical effects.

## Data Availability Statement

Data for this study from the CARE Consortium are publicly available from the National Institute of Health (NIH) Federal Interagency Traumatic Brain Injury (TBI) Research (FITBIR) Informatics System (https://fitbir.nih.gov).

## Ethics Statement

The studies involving human participants were reviewed and approved by Medical College of Wisconsin Institutional Review Board and the Human Research Protection Office (HRPO). The patients/participants provided their written informed consent to participate in this study.

## Author Contributions

BB designed and conceptualized study, analyzed data, interpreted data, and drafted the manuscript for intellectual content. SB analyzed data, interpreted data, and drafted the manuscript for intellectual content. LE major role in the acquisition of data, analyzed data, and revised the manuscript for intellectual content. DH analyzed data and the manuscript for intellectual content. AM drafted and revised the manuscript for intellectual content. JH analyzed data and revised the manuscript for intellectual content. SB and TM designed and conceptualized study, major role in the acquisition of data, and revised the manuscript for intellectual content. MM designed and conceptualized study, major role in the acquisition of data, interpreted data, and drafted the manuscript for intellectual content. TM designed and conceptualized study, major role in the acquisition of data, analyzed data, interpreted data, and drafted the manuscript for intellectual content. All authors approved the final manuscript as submitted and agree to be accountable for all aspects of the work.

## Conflict of Interest

The authors declare that the research was conducted in the absence of any commercial or financial relationships that could be construed as a potential conflict of interest. The reviewer, BA, declared a past co-authorship with several of the authors SB, TM, and MM to the handling editor.
